# Alumni Meeting

**Published:** 1867-08

**Authors:** 


***Alumni Meeting.***

Prof. Rea informs us that his suggestion of a meeting of the Alumni of Rush College, to inaugurate the opening of the new college edifice, is being responded to with great enthusiasm. The meeting will be held early in October, and a sufficient number have already pledged themselves to be present to secure a highly interesting session. There are more than a thousand of the Alumni, besides those who have attended the lectures and not yet received its honors.

"Old Rush" proposes to extend a general amnesty to those who have deserted her, temporarily, because of her inability to afford them shelter, aid and comfort. Hereafter there will be no occasion for filial wanderings.

The little schools who endeavor to make up for paucity of students by adding to the roll of professors, (and some of them positively exhaust the graduating class of one session to make up a Faculty for the next,) are jubilating over certain action of a, so called, Teacher's Convention, which seemingly gave them warrant for their doings; but the profession understand the "dodge," and will not be deceived.

The great schools of the country propose to regulate their internal affairs in their own way. They propose to be in advance in the *practical*, and to leave the dreams of theorists to prove their nothingness by results.

They propose to teach adult *men*, who crowd their halls, *for a purpose* as men, and not like idle school-boys, whining over their tasks.

To its Alumni, of whom "RUSH" is proud, "*Greeting!*"

